# Optimizing Tumor Microenvironment for Cancer Immunotherapy: β-Glucan-Based Nanoparticles

**DOI:** 10.3389/fimmu.2018.00341

**Published:** 2018-02-26

**Authors:** Mei Zhang, Julian A. Kim, Alex Yee-Chen Huang

**Affiliations:** ^1^Department of Biomedical Engineering, Case Western Reserve University, Cleveland, OH, United States; ^2^Case Comprehensive Cancer Center, Cleveland, OH, United States; ^3^Seidman Cancer Center, University Hospitals, Cleveland, OH, United States; ^4^Division of Surgical Oncology, Department of Surgery, Case Western Reserve University School of Medicine, Cleveland, OH, United States; ^5^Division of Pediatric Hematology-Oncology, Department of Pediatrics, Case Western Reserve University School of Medicine, Cleveland, OH, United States

**Keywords:** cancer immunotherapy, tumor microenvironment, immune modulator, beta-glucan, beta-glucan-based nanoparticle

## Abstract

Immunotherapy is revolutionizing cancer treatment. Recent clinical success with immune checkpoint inhibitors, chimeric antigen receptor T-cell therapy, and adoptive immune cellular therapies has generated excitement and new hopes for patients and investigators. However, clinically efficacious responses to cancer immunotherapy occur only in a minority of patients. One reason is the tumor microenvironment (TME), which potently inhibits the generation and delivery of optimal antitumor immune responses. As our understanding of TME continues to grow, strategies are being developed to change the TME toward one that augments the emergence of strong antitumor immunity. These strategies include eliminating tumor bulk to provoke the release of tumor antigens, using adjuvants to enhance antigen-presenting cell function, and employ agents that enhance immune cell effector activity. This article reviews the development of β-glucan and β-glucan-based nanoparticles as immune modulators of TME, as well as their potential benefit and future therapeutic applications. Cell-wall β-glucans from natural sources including plant, fungi, and bacteria are molecules that adopt pathogen-associated molecular pattern (PAMP) known to target specific receptors on immune cell subsets. Emerging data suggest that the TME can be actively manipulated by β-glucans and their related nanoparticles. In this review, we discuss the mechanisms of conditioning TME using β-glucan and β-glucan-based nanoparticles, and how this strategy enables future design of optimal combination cancer immunotherapies.

## Current Status of Cancer Immunotherapy

Cancer immunotherapy was first reported using Coley’s toxin to provoke patient’s own immune responses (1). Soon after that, many studies have been performed. Preclinical animal studies have identified tumor-specific antigens and the direct killing of tumor cells using T cells specific for these antigens (2, 3). Some of these findings were recapitulated in humans and used to develop cancer immunotherapies based on provoking tumor-antigen-specific T-cell responses (4, 5). However, such immunotherapeutic approaches showed only limited clinical effect and have yet to become clinical standard of care. Subsequently, studies on tumor-induced immune suppressive mechanisms have identified multiple suppressor cell populations and inhibitory molecules, all of which exert critical regulatory roles in tumor generation and progression (6–8). These studies resulted in the development of first cancer immunotherapy agent anti-CTLA-4 monoclonal antibody (mAb), ipilimumab, which blocks the negative co-stimulatory signaling as a result of binding between cytotoxic T-lymphocyte-associated protein 4 (CTLA-4) and B7.1 (9). Clinical trial with ipilimumab demonstrated greater durable responses in advanced melanoma patients as compare to conventional therapy; therefore, it was approved by the US Food and Drug administration (FDA) for the treatment of unresectable or metastatic melanoma in 2011 (10). Thereafter, anti-PD-1 mAb, nivolumab, which blocks programmed death-1 (PD-1) binding to PD ligand 1 (PD-L1), was approved for the treatment of unresectable melanoma in 2014 (11). The FDA subsequently expanded the approved use of nivolumab to patients with advanced squamous non-small cell lung cancer (NSCLC) with progression on or after platinum-based chemotherapy in 2015 (12). In May 2017, the FDA granted approval to pembrolizumab (anti-PD-1 mAb) for locally advanced or metastatic urothelial carcinoma, the most common type of bladder cancer, which progressed during or after platinum-containing chemotherapy or within 12 months of neoadjuvant or adjuvant chemotherapy. The FDA also granted accelerated approval to pembrolizumab for patients with locally advanced or metastatic bladder cancer who are not eligible for cisplatin-containing chemotherapy (13).

The clinical successes of ipilimumab and nivolumab have encouraged the development of therapies targeting additional immune checkpoint molecules. As the activation of T cells is controlled by a balance of co-stimulatory molecules that act positively in concert with T-cell receptor (TCR) signaling, and co-inhibitory molecules, including immune checkpoint molecules, which negatively regulate TCR signaling (14), multiple agents are being developed to target co-stimulatory molecules including CD27 (15), CD28 (16), inducible T-cell co-stimulator (17), 4-1BB (18), OX40 (19), and co-inhibitory molecules including (CTLA-4) (20), PD-1 (21), PD-L1 (22), T-cell immunoglobulin and mucin domain-containing molecule-3 (23) and lymphocyte-activation gene 3 (24). These agents aim to repair immune balance at the immune checkpoint in order to promote the activation and generation of tumor-specific effector T cells. Currently, ipilimumab (mAb targeting CTLA-4), nivolumab, pembrolizumab, and pidilizumab (mAbs targeting PD-1), as well as MDX-1105, MPDL3280A, MEDI4736, and MSB0010718C (mAbs targeting PD-L1) are being tested in multiple clinical trials targeting metastatic melanoma, advanced NSCLC metastatic solid tumors, and hematological malignancies with variable clinical outcomes (25–27).

In addition to immunotherapies targeting immune checkpoint molecules, therapies targeting immunosuppressive regulatory T cell (T_reg_) are also being pursued. The CD25^+^CD4^+^FoxP3^+^ T_reg_ cells are potent suppressors of antigen-specific or non-specific tumor immunity (28), with FoxP3 is a master gene that controls the differentiation of CD25^+^CD4^+^ T-cell and its functions (29). FoxP3^+^ T_reg_ population are heterogeneous and have been classified into three sub-populations: CD45RA^+^FoxP3^low^ (naïve T_reg_), CD45RA^−^FoxP3^high^ (effector T_reg_) and CD45^−^FoxP3^low^ (non-T_reg_). Available data suggest that effector T_reg_ (e-T_reg_) has the highest suppressive activity among these subpopulations (30). Since CTLA-4 expression is one of the major suppressive mechanisms mediated by T_reg_, mAb targeting CTLA-4 was developed to inhibit the suppressive activity of CTLA4 on T cell activation (31, 32). Agents targeting GITR, OX40, and CD15S are also being investigated, as these molecules are selectively expressed on e-T_reg_ and have functional roles (33, 34). Some conventional chemotherapeutic drugs such as cyclophosphamide (35), paclitaxel (36), gemcitabine (37), and docetaxel (38) are found to arrest cell cycle of T_reg_, and are being tested in clinical trials to assess their effect on reducing T_reg_ number and function to improve antitumor immunity in cancer patients.

Another area of immune-modulatory therapy is the targeting of tumor-associated macrophages (TAMs) and myeloid-derived suppressor cells (MDSCs). The presence of TAMs are associated with tumor progression, survival, angiogenesis, and the suppression of antitumor immunity (39), while MDSCs are heterogeneous populations that not only suppress immune activity but also support tumor growth and progression (40). Conventional chemotherapeutic drugs such as gemcitabine, 5-fluorouracil, docetaxel, doxorubicin, paclitaxel, and sunitinib exhibit selective inhibitory effect on MDSCs due to higher proliferative activity of MDSCs as compared to effector T-cells or natural killer (NK) cells (41). In addition, low-dose paclitaxel has been observed to promote MDSC differentiation into dendritic cells (DCs) (42). Doxorubicin exhibits an ability to induce MDSC apoptosis by enhancing the production of ROS (43). Docetaxel can promote MDSC differentiation into M1 macrophages by blocking Stat3 phosphorylation (44). Sunitinib inhibits VEGF and/or c-KIT-mediated signaling pathway in MDSCs (45). Precise molecular mechanisms of these drugs affecting MDSC function remain to be fully investigated.

Other potent immunotherapeutic approaches include chimeric antigen receptor (CAR) T-cell therapy (46), TCR cellular therapy (42), and tumor-associated antigen cancer vaccines (43). The first CAR-T cell therapy has been approved by the FDA in August of 2017. While there is much excitement surrounding CAR-T cell therapy, the broad application of this approach has been limited by the availability of suitable targets in various cancers, cost of making individualized engineered T cells, and the availability of autologous T cells for therapy production, especially in heavily pretreated patients or those with leukemic relapses soon after myeloablative bone marrow transplantation.

In summary, mAbs against immune checkpoint molecules demonstrated superior clinical effects compared with conventional therapy for tumors including advanced melanoma, NSCLC, and renal cancer. However, there is room for greater improvement on the clinical efficacy of this and other aforementioned immunotherapeutic approaches.

## Additional Tumor Microenvironment (TME)-Modifying Strategies to Enhance Cancer Immunotherapy

Although ongoing clinical immunotherapies including antibodies targeting immune checkpoint molecules can result in durable responses in some otherwise therapy-refractory cancer patients, only a minority of all cancer patients benefit from these advances. Data suggest that positive responses rely on dynamic enhancing interactions between tumor cells and immune cells within the TME (47). TME plays an important role to either dampen or enhance immune responses in a context-dependent manner. As the understanding of TME increases, strategies are emerging for changing the TME from an immunosuppressive one toward one that supports the enhancement of antitumor immunity.

Current strategies using immune modulators to manipulate TME include the following. (1) The use of immune checkpoint inhibitors as immune modulators to enhance endogenous antitumor responses in TME (48, 49). (2) Targeting regulatory cells in TME. Relevant regulatory cells within TME include T_reg_, TAMs (M2-type macrophages) and MDSCs. Suppressive mechanisms employed by these cells involve secretion of cytokines (e.g., IL-10 and TGF-β), enzymes (e.g., arginase, NOS, and IDO), and expression of inhibitory receptors (e.g., CTLA-4 and PD-L1). Targeting these regulatory cells and their suppressive mechanisms to alter immunosuppressive nature of TME can be anticipated to enhance immunotherapy (50–59). (3) Modifying the chemokine profile of TME. Cellular composition of tumors is heavily influenced by the chemokine profile in the TME. Various types of leukocytes are attracted in response to specific chemokines. Therefore, manipulation of the chemokine profile can potentiate antitumor activity by altering immune cellular components (60–62). (4) Modulating danger signals: toll-like receptors (TLRs). TLR agonists can trigger broad inflammatory responses that elicit rapid innate immunity and promote activation of the adaptive immune reaction. Oncolytic viruses are highly immunogenic pathogens capable of stimulating TLR, and because they infect or replicate predominantly in tumor cells, much of their activity is localized to tumors (63, 64). (5) Manipulating cytokines in TME. The cytokine content of the microenvironment can tip the balance between immunosuppressive and immune-activating factors within tumors. Many types of immunotherapy benefit from co-administration of cytokines, but delivery of these cytokines is often systemic making it difficult to distinguish between contributions from microenvironment modification and systemic immune modulation. Several studies have directed cytokines specifically to tumors using engineered cytokine-producing T-cells or targeted nanoparticle system. They demonstrate observable changes in the TME and increased the efficacy of additional immunotherapeutic agents (65, 66). (6) The use of virus-like particles (VLPs) as immune modulator of TME. VLPs refer to the spontaneous organization of viral coat proteins into the 3D structure of a particular virus capsid. They are generally in the 20–500 nm size range but lack virus nucleic acid such that they are reported to be non-infectious. It has been reported in a recent study that a VLP system derived from cowpea mosaic virus possess inherent immunogenic properties that stimulate immune responses against tumor cells in preclinical mice models, and protective immunity in the models was associated with leukocyte recruitment, increased antigen-processing capabilities, stimulation of T and B lymphocytes (67). However, VLP-mediated immunomodulation appears to be non-specific and the exact cellular and molecular mechanisms remain to be defined.

The above strategies can be used to modify the TME to support additional immunotherapies (Table 1). However, the majority of these studies were limited to preclinical investigations in mice. Most clinical studies involving combination of immune modulators and immunotherapeutic agent(s) are still in early stages of clinical trials, and the precise mechanisms remains to be established. We believe it is crucial to carefully evaluate immune modulators with respect to the specificity, immune-potentiating capability, and potential toxicity prior to commencing clinical trials.

**Table 1:** Examples of strategies of modulating tumor microenvironment (TME) to enhance cancer immunotherapy.

Strategies to modulate TME		Immune modulator of TME	Cancer immunotherapy	Modulatory effect within TME	Antitumor effect	Reference
1. Use immune checkpoint inhibitors to modulate TME		mAbs blocking programmed death-1 and CTLA 4	mAbs targeting PDL-1 plus -irradiated B16 tumor cell vaccine expressing Flt3L	Increased T cell infiltration into tumor, IFN-g production, ratio of effector T-cells to myeloid-derived suppressor cells (MDSCs)	Rejection of B16 tumor	(48)
		mAbs blocking immune checkpoint molecule CD73 (CD73 inhibits T cell adhesion to endotheial cells and localization to tumors)	ACT of tumor-specific cytotoxic T lymphocytes (CTLs)	Restored T cell adhesion and homing, enhance effector T-cell accumulation in tumor	Delayed tumor growth and enhanced survival of mice bearing B16 tumor	(49)
2. Targeting regulatory cells within TME	2.1. Blocking differentiation of regulatory cells within TME	Abs blocking CCL1	CpG immunotherapy	Neutrlization of the *de novo* conversion of T_reg_	Complete tumor rejection in mice bearing TUBO tumors	(50)
	2.2. Blocking recruitment of regulatory cells to TME	Abs blocking CCL2 (CCL2 is chemoattractant for myeloid suppressor cells)	ACT	Increased infiltration of tumor-specific T cells	Delayed tumor growth and enhanced survival of mice bearing EG7 or MCA-203 tumors	(51)
		Small molecule antagonist of CCR4 (CCR4 helps to induce CCL-17 and CCL22-mediated T_reg_ recruitment)	ACT	Increased infiltration of effector CD8^+^ T cells	Tumor growth inhibition	(52)
	2.3. Blocking immunosuppressive enzymes secreted by regulatory cells	*N*-hydroxy-l-Arg (NOHA) (NOHA targets enzyme (arginase)-expressing M2-type macrophages)	OX40 immunotherapy	Reduction of MDSCs and increase of tumor-infiltrating specific CTLs	Increase survival of mice bearing sarcoma	(53)
		1-methyl-tryptophan (IDO inhibitor) (IDO is enzyme secreted by regulatory cells)	IL12 + GM-CSF microspheres	Transient reduction of Treg, and increased ratio of CD8^+^ T-cells to T suppressor cells	Tumor rejection in mice bearing metastatic 4T1 tumors	(54)
	2.4. Depleting regulatory cells	Clodronate encapsulated in liposomes (deplete macrophages)	Antiangiogenic immunotherapy (anti-VEGF or anti-CD137 Abs) ACT or cancer vaccine	Reduction of CD11b^+^ tumor-associated macrophages	Delayed tumor growth regression of tumor	(55, 56)
		mAbs targeting Gr-1 (deplete MDSCs)		Reduction of MDSCs		
	2.5. Reprogramming immunosuppressive cells	Chemotherapy	Anti-CD40 Abs	Redirect infiltrating macropages to antitumor potential	Remarkable survival in both mice and patients with pancreatic carcinoma	(57)
		IDO inhibitor	cancer vaccine	Conversion of T_reg_ to a Th17 phenotype with marked enhancement of CD8^+^ T-cell activation	Delayed tumor growth and improved survival of mice bearing B16F10 tumors	(58)
		Gemcitabine	Recombinant adenovirus expressing the tumor-associated antigens Her-2 and anti-GITR antibody	Revert *in vivo* T_reg_ immunosuppressive activity	Therapeutic effectiveness against pre-existing tumor	(59)
3. Modifying chemokine profile of TME		Adenovirus- or plasmid-encoded CXCL10 or XCL1 [chemokines attract CD8^+^ T cells, natural killer (NK) cells and monocytes]	ACT of CTLs or DC vaccines	Increased infiltration of CD4^+^, CD8^+^, and NK cells	Tumor regression or eradication	(60)
		Oncolytic viruses encoding CCL5 or CCL2	Tumor-lysate-pulsed dendritic cells (DCs)	Significant increase of tumor infiltration of CD8^+^ T cells	Eradication of tumors in mice bearing neuroblastoma	(61)
		Intratumoral injection of CCL21 or CCL16 (chemokines attract DCs and macrophages and T cells)	CpG immunotherapy	Infiltration of CD4^+^ T cells and DCs	Eradication of tumors in mice bearing tumors of TSA, 4T1, and MC38	(62)
4. Modulating inflammatory mediators and toll-like receptor		Oncolytic vaccinia virus	Anti-CD137 agonist Abs	Increased infiltration of CD8^+^, NK cells and neutrophils	Tumor eradication in mice bearing AT3 tumors	(63)
		HSV-TK retrovirus adhering to cells	ACT of CTLs + gancyclovir + lymph-depletion	Maximum number of T cells in tumor occurred at 72–96 h	Improved survival of mice bearing B16-OVA	(64)
5. Manipulating cytokines in TME		IL12 transgene in T-cells	ACT of CTLs + lymph-depletion	Reversed suppression of MDSCs and other suppressive myeloid cells in tumors	Improved survival of mice bearing B16 tumors	(65)
		TGF-b inhibitor in liposome gel	IL-2	Increased infiltration of NK cells and activated CD8^+^ T-cells	Improved survival of mice bearing B16F10 tumors	(66)
6. Virus-like particles (VLPs) immune modulator of TME		VLP from cowpea moaic virus		Increased recruitment of antitumor neutrophils, increased activation of T and B cells		(67)

Our team has focused on the development of β-glucan and β-glucan-based nanoparticles as immune modulators of TME. The β-glucan-based molecules are derived from natural resource and the safety profile has been well demonstrated. These molecules adopt pathogen-associated molecular pattern (PAMP), which has known mechanisms of targeting specific receptors on immune cell subsets. The remainder of this review will discuss the use of β-glucan and β-glucan-based nanoparticles as immune modulators of TME, their specificity, potential benefit, their advantages over other types of immune modulators, and future therapeutic applications. We will also review how β-glucan mediate changes in TME, and how this change enables the design of optimal combination cancer immunotherapies.

## β-Glucan and β-Glucan-Based Nanoparticles as Effective Immune Modulator to Enhance Cancer Immunotherapy

Polysaccharides, also known as β-glucans, can be extracted from the cell walls of natural resources such as plant, fungi, and bacteria. They are biomolecules that can adopt pathogen-associated molecular patterns and can modulate host immune responses *via* priming and/or stimulating innate immune cells such as macrophages, neutrophils, and granulocytes (68). Both *in vitro* and *in vivo* studies have suggested that Dectin-1, complement receptor 3 (CR3), and TLR-2/6 are critical receptors mediating such priming and stimulation of innate immune cells by β-glucans (69). Binding of β-glucan on these receptors can trigger immune cells including macrophages, neutrophils, monocytes, NK cells, DCs, as well as T cells (70, 71). Recent preclinical mouse studies have demonstrated that the systemic administration of certain β-glucans could effectively manipulate TME, resulting in significant reduction of primary tumor growth and distant metastases (72). These results suggest that β-glucan molecules are potential immune modulator that can manipulate innate and adaptive immune responses within the TME and improve clinical responses of current cancer immunotherapies. As compared to the protein-, peptide-, virus-, and virus-like-particle-based immune modulators, β-glucan has several advantages: (1) β-glucan is non-immunogenic molecule due to an absence of the protein and peptide components so as not to cause non-specific immune activation; (2) β-glucan has been demonstrated to be non-toxic. A high dose up to 10 mg/kg is well tolerant *in vivo* with no adverse effect observed; (3) immunomodulatory effect of β-glucan is specific because it mediates immune potentiating activity on immune cells *via* specific surface receptors (discussed below); (4) β-glucan contains multiple aldehyde and hydroxyl groups, which provide opportunities for structural modification, improved physiological property, and construction of β-glucan-based nanoparticle system with capacity for carrying high payload of immune-modulating agents. We will review below the specific immune potentiating mechanism of β-glucan and the potential clinical benefit, and discuss the therapeutic development of β-glucan and the related nanoparticles in combination with additional cancer immunotherapy for best possible clinical outcome.

### Potential Routes of Administration for β-Glucan

The potential route by which external β-glucans enter the blood stream is a very important factor in the eventual development of the β-glucan class of molecules as effective immune modulators. The human body lacks the enzyme(s) capable of digesting β-glucans. It has been well documented that β-glucans are resistant to digestion in the gastrointestinal tract (48) and require the bacterial fermentation process occurring within the large intestine (73). Additional studies over the past 40 years have examined the potential routes by which some β-glucans can enter the bloodstream to exert their biological activities. Rice et al. demonstrated that after oral administration, β-glucans can directly interact with the gastrointestinal mucosa and are then transferred to the general circulation. These studies implicated a process of β-glucan internalization by epithelial cells (possibly M cells), macrophages and DCs, which then rapidly entered the systemic circulation (74). Chan et al. documented uptake and internalization of β-glucans from the gut by macrophages, which then circulated in the blood and released β-glucans throughout the body (71). Sandvik et al. reported a total plasma β-glucan content of ~30 ng following a 14-day oral administration consisting of 5–6 mg β-glucans per day (75). Bioavailability characterization of several soluble and insoluble β-glucans revealed that the ratio of measurable glucan entering the blood to total orally glucan intake ranged from 0.5 to 5% depending on the molecular weight (Mw) and surface charge (74–77). Based upon results of these studies, oral administration is deemed as an effective route for β-glucans to enter the blood stream, and the actual efficiency of β-glucans entering blood circulation varies according to the distributions of their Mw, polydispersity, side chain branching, root mean square radius, and solution conformation.

On the other hand, studies involving systemic administration (i.v. or i.p.) of β-glucans have also been conducted. These results showed that both i.v. and i.p. administration could result in higher blood β-glucans levels with active biological activities. The half-lives of i.v. or i.p. administered β-glucans—typically on the order of hours to 10 h—were dependent on the chemical structure, Mw, side chain branching, soluble and insoluble forms, surface charge, etc. (75, 78).

### β-Glucan Sources, Structure, and Active Moiety

β-glucans are polymers composed of glucose. They are cell-wall component of plants and microorganisms such as oat, barley, mushroom, seaweed, some bacteria, and yeast. Oat and barley β-glucans exhibit linear chain structure with large regions of both β-(1–3) and β-(1–4) linkages (hereafter referred to as BG34) (79, 80). Mushroom and fungal β-glucans have β-(1–3) backbone grafted with β-(1–6) side chains of various sizes and numbers and are, therefore, referred to as BG36 (81, 82). β-glucans from different resources and with different structures exhibit different solubility, physiological properties, and biological activities (83). The molecular size and complexity of β-glucans are also important for biological activity because they can influence the interaction of β-glucan with human and murine monocytes and macrophages (84, 85).

**Figure 1:**
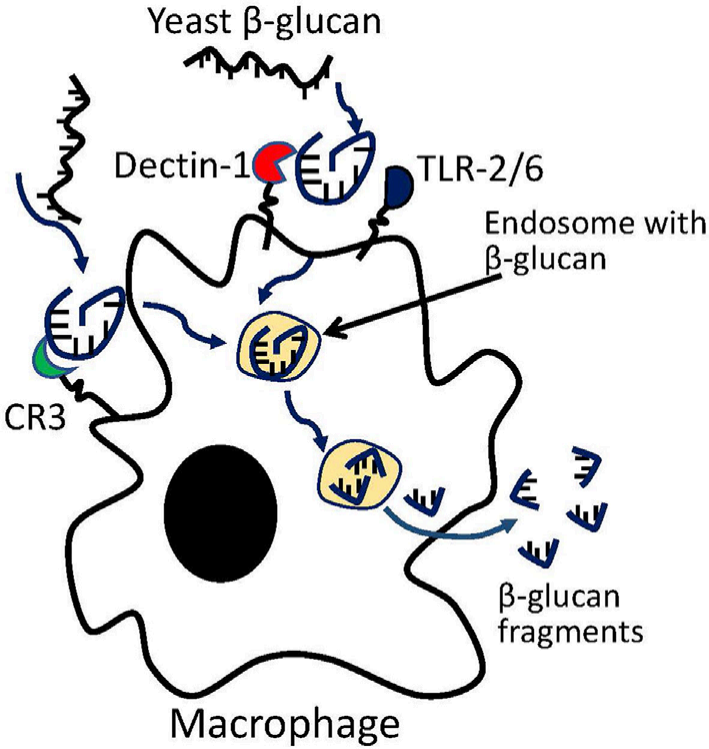
Macrophage processing of yeast β-glucans into small β-glucan fragments.

Previous studies on yeast β-glucans have suggested that the β-(1–3) glucan fragments may be the active moiety (86). *In vitro* coculture of primary macrophages with yeast β-glucan resulted in macrophage digestion of glucan into β-(1–3) glucan fragments (Figure 1). The digested fragments can be detected in macrophages as early as 3 days following coculture, but a more complete processing of parent β-glucan requires ~14 days. The fragments were found to prime effector cells such as macrophage, neutrophil, and granulocytes for antitumor efficacy (87). *In vivo* studies have shown that β-(1–3) glycosidic backbone of yeast glucan could not be digested in stomach so that most glucans enter the proximal small intestine, where the yeast glucans were captured by macrophages and digested into small fragments within macrophages. Glucan fragments could be transported by macrophages to bone marrow and the endothelial reticular system in mice, suggesting that the β-(1–3)-glucan fragments may be the active moiety of yeast glucan (88). However, whether these fragments compose the active moiety of other glucan subtypes remains to be examined. β-glucans with different structures, solubility, Mws, and routes of administration have shown variable immune potencies. Furthermore, the biologically active moieties of other glucan structures may be different from that from yeast and need to be further studied.

### Mechanisms of β-Glucan-Mediated Antitumor Immune Response and Its Therapeutic Benefits

β-glucans of different resources, structures, and components mediated antitumor immune responses in different manners. For instances, zymosan, an insoluble yeast-derived β-glucan consisting of BG36 glucan and protein complexes, mediates immune responses by increasing the number and function of macrophages as well as activating the complement system (89). Lentinan, a mushroom-derived BG36 glucan with poor water solubility, mediates antitumor immune response by increasing the lymphokine-activated killer cell activity and NK cell activity (90). A particulate β-glucans derived from yeast mediates antitumor immune responses by inducing pro-inflammatory cytokine secretion and stimulating innate immune effector cell activation (70). These observations support the view that β-glucan-based molecules act through different mechanisms to stimulate antitumor immune responses.

*In vitro* and *in vivo* preclinical studies on a particulate yeast-derived BG36 glucan-protein complex implicates the dectin-1 pathway as playing a very important role in the glucan-protein complex’s ability to mediate antitumor immune responses. *In vitro* coculture of macrophages with the particulate yeast-derived BG36 glucan-protein complex has resulted in the activation of the macrophages, which promoted Th1 and cytotoxic T-lymphocyte priming and differentiation. In support of this, glucan-mediated macrophage activation was completely abolished in the presence of anti-dectin-1 blocking Abs. Oral administration of the particulate yeast-derived BG36 glucan-protein complex resulted in downregulating immunosuppressive cells such as T_reg_ and MDSCs, and led to delayed tumor progression in wild-type mice. However, the striking antitumor effects were completely abrogated in dectin-1 knockout (KO) mice using the same treatment schedule with β-glucan (91–94). In addition, MDSCs that exhibited high dectin-1 expression could also be modulated by the particulate yeast-derived BG36 glucan-protein complex. *In vitro* coculture of MDSCs with the glucan-protein complex has resulted in the reduction of c-jun-molecule-dependent expression of nuclear factor I-A (NFIA) of granulocytic-MDSCs (G-MDSCs). Note that NFIA is an integral transcriptional component of myeloid cells and plays critical role for myeloid differentiation and lineage commitment, and the NFIA knockdown can alter the suppressive function of G-MDSCs, promote the antitumor immune responses, and delay tumor progression in mice (92). These *in vitro* and *in vivo* observations suggest that the dectin-1 pathway play a very important role for the particulate yeast-derived β-glucan-protein complex to interact not only with macrophages but also with G-MDSCs to reduce the immunosuppressive function and potentiate the development of antitumor immune responses.

**Figure 2:**
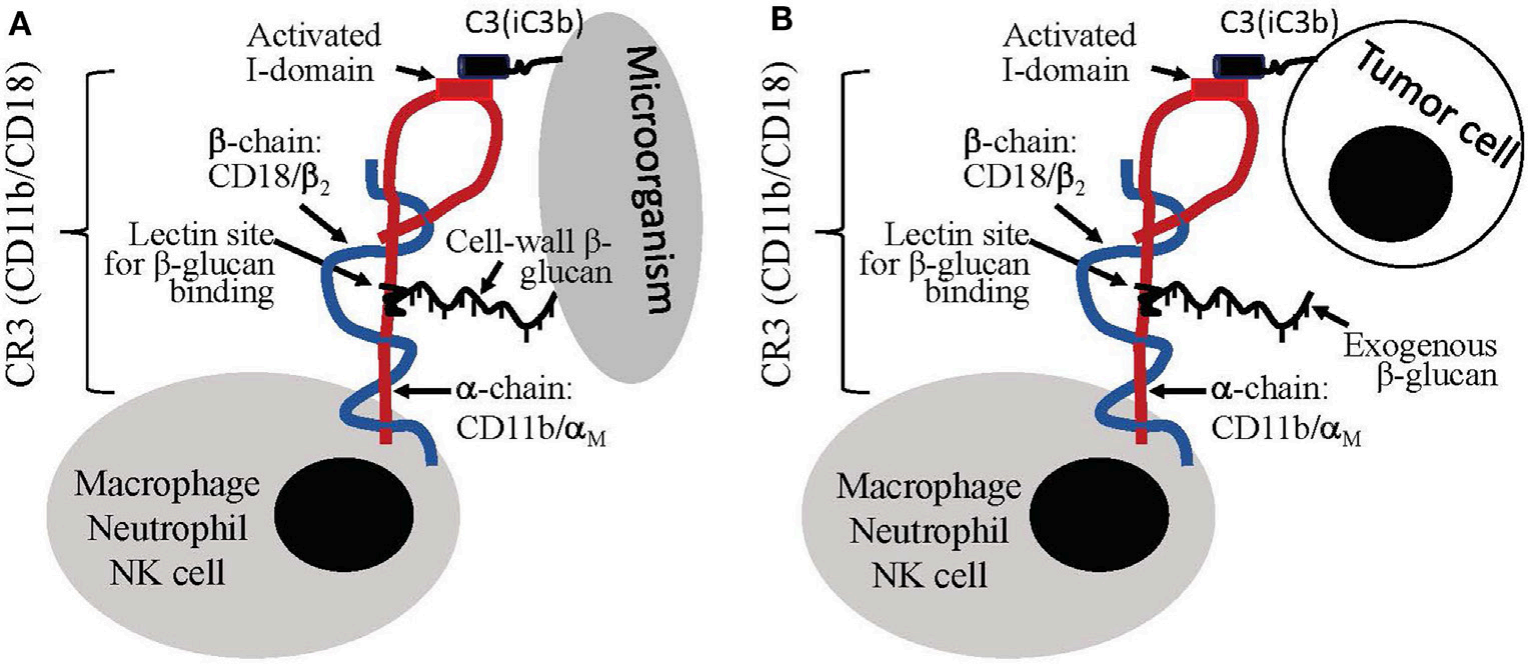
Leukocyte priming/activation induced by β-glucans. **(A)** Cell-wall β-glucans of microorganism can induce dual binding, i.e., CD11b-β-glucan binding and CD18-iC3b binding, to leukocytes, which stimulate leukocytes for complement receptor 3 (CR3)-depedent cellular cytotoxicity (CR3-DCC). **(B)** Tumor cells lack of β-glucans do not induce dual binding to leukocytes; however, the introduction of exogenous β-glucan can create dual-binding of leukocyte to iC3b-positive tumor cells to stimulate CR3-DCC for the destruction of the target.

Distinct from particulate yeast-derived BG36 β-glucan-protein complex, a soluble β-glucan fraction derived from yeast was shown to mediate antitumor immune responses through a specific activation of CR3 on leukocytes. CR3 is a α_Mβ2_ integrin family glycoprotein, which is widely expressed on the surface of monocytes, macrophages, granulocytes, NK cells, and subsets of DCs and B cells. One of the critical biological function of CR3 is the CR3-dependent cellular toxicity (CR3-DCC), which is critical for the killing and clearance of microorganisms. Microorganism cell-wall is composed of β-glucan, which can bind to CD11b subunit of CR3 on leukocytes. This binding results in the activation of I-domain on the CD18 subunit of leukocyte CR3. The binding also results in complement activation and iC3b deposition on the surface of microorganisms. The ligation between CD11b-β-glucan-binding and the CD18 (I-domain)-iC3b-binding initiates killing based on a CR3-mediated mechanism, i.e., CR3-DCC, against the microorganisms. In other words, the killing of microorganism by leukocytes (mainly macrophages) requires the ligation of two binding sites within CR3 (Figure 2A) (95–97). Different from microorganisms, mammalian cells including tumor cells do not have β-glucan molecules on the surface. Therefore, although tumor cells could be coated with iC3b molecules, the binding of iC3b to CD18 subunit of CR3 on leukocytes is not sufficient to induce the leukocyte to kill the target (tumor cells) due to a lack of β-glucan-binding to CD11b on leukocytes (Figure 2B).

Based upon this knowledge, antitumor therapy using soluble yeast-derived β-glucan was proposed and evaluated. *In vitro* studies demonstrated that β-glucan co-culture with leukocytes such as macrophage and neutrophil could trigger the binding of soluble β-glucan to CD11b on leukocytes, which resulted in the activation of I-domain of CD18 and its binding to iC3b-positive tumor cells. In response to iC3b-positive tumor cells, β-glucan-primed leukocytes produced cytokines including TNF-α, IFN-γ, IFN-α, and IL-6, which are associated with tumor cell destruction. Interestingly, β-glucan-primed leukocytes did not produce cytotoxic cytokines in response to iC3b-negative cells, suggesting that β-glucan has the potential to mediate specific antitumor effect without undesirable cytotoxicity to bystander cells (98).

To bolster this finding, the CR3-dependent antitumor activity of the soluble yeast-derived β-glucan has been demonstrated in a variety of murine tumor models and human tumor xenografts. The highly purified, low Mw and soluble yeast-derived BG36 β-glucan was intravenously (i.v.) administered to various tumor-bearing mice strains. Tumor regression required the pre-existence of antitumor Abs. In mice with low naturally derived Ab titers, antitumor efficacy of the yeast-derived β-glucan was significantly compromised (99). Indeed, β-glucan therapy completely failed to slow down tumor growth in Ab-deficient SCID mice. However, the β-glucan-mediated antitumor efficacy could be restored by passive immunization with either purified natural Ab in SCID mice or purified antitumor mAbs in mice with low naturally derived antitumor Ab titers (100). β-glucan therapy in combination with antitumor mAbs could enhance both tumor regression and long-term survival as compared to treatment with antitumor mAb or β-glucan therapy alone (101). More importantly, β-glucan-mediated antitumor efficacy was abrogated in either C3-deficient or CR3-deficient mice (102). Altogether, these results showed that soluble yeast-derived BG36 glucan could prime leukocytes *via* CR3 to induce specific antitumor immune responses *via* Ab-opsonization and iC3b-coating of tumor cells.

Complement receptor 3-dependent antitumor efficacy has also been demonstrated in tumor-bearing mice treated with soluble BG34 β-glucans derived from barley or oat. Daily oral administration of barley BG34 glucan in combination with an antitumor Ab, mAb14G2a, resulted in significant tumor regression and prolonged survival of neuroblastoma-bearing mice as compared to mice treated with mAb alone. This barley BG34 glucan-mediated rejection was significantly diminished in CR3-deficient mice, suggesting leukocyte CR3 as playing a critical role for soluble barley BG34 glucan-mediated antitumor immune responses (103). In our studies, we observed that oat-derived BG34 β-glucan of a specific Mw range could effectively enhance phagocytosis activity of macrophages *via* CD11b subunit of CR3. *In vitro* coculture of fluorescence (FITC)-conjugated BG34 glucans of various Mw with bone marrow-derived macrophages showed that BG34 of Mw less than 500 kD mediated effective uptake by macrophages within 6 h, and these glucan-treated macrophages showed significantly enhanced phagocytosis of latex beads. Macrophage uptake of BG34 was significantly inhibited in the presence of CD11b blocking Ab, but not anti-dectin-1 Ab, suggesting CD11b as playing a very important role for BG34 glucan-mediated activation of macrophages. Coculture of FITC-BG34 glucans with MDSCs showed that BG34 glucan of ~200 kDa could trigger direct binding to CD11b-overexpressing MDSCs (104). Furthermore, daily intraperitoneal administration of oat-derived 200 kDa BG34 glucans resulted in a reversal of TME to a pro-immunogenic one, consisting of M1-type activation of macrophages, activation of DCs, and the induction of cytokines/chemokines such as IFN-γ, TNF-α, CXCL9, CXCL10, PDL-1, and IRF-1 that are associated with T cell infiltration and tumor eradication. The oat-derived BG34 glucan treatment also resulted in a significant increase of activated T cells in the tumor-draining lymph nodes (LNs). BG34-induced alteration in TME and LN immune signatures resulted in regression of established primary and metastasis of B16F10 melanoma model in C57BL/6 mice. Such BG34-induced tumor regression was not observed in the CD11b KO mouse model (105). Results of these studies suggested that soluble BG34 glucans have a great potential to prime immune cells and enable them to mediate innate and adaptive antitumor immune responses.

In addition to the yeast-, oat-, and barley-derived glucans, bacteria-derived BG36 β-glucan, Curdlan, has shown beneficial modulatory effects on adaptive immunity. Coculture of Curdlan with T_reg_ *in vitro* resulted in the conversion of T_reg_ into Th17 effector cells. Preclinical studies *in vivo* showed that oral administration of Curdlan could induce both Th1 and Th17 differentiation in mice bearing 4T1 mammary tumors. Curdlan treatment could also improve CD8^+^ T cell priming *via* its potent adjuvant effect. The conversion from T_reg_ cells into Th17 effector cells by β-glucan remains to be confirmed in *in vivo* tumor models. The discovery that β-glucan conversion of T_reg_ into Th17 effector cells may lead to the development of innovative cancer immunotherapies with enhanced efficacy (106).

Purified forms of certain β-glucans from yeast, fungus, bacteria, and plants have demonstrated defined immunomodulatory effects in tumor-bearing hosts. Accumulating studies have provided increasing evidence to support the view that certain species of β-glucans interact with specific immune cell subpopulations to enhance anticancer immunity (76, 107–110). One such mechanism is the effect of β-glucan interactions with DCs and the resulting immune responses. Studies have shown that selective cytokine responses can be produced by stimulating DCs depending on the nature of interacting species of β-glucans (chemical structure, Mw, and surface charge) (111). Systemic administration of fungal β-glucans was shown to stimulate measurable plasma levels of TNF-α, and administration of fungal β-glucans in combination with TLR antagonists resulted in a significant increase of IL-12p70 in plasma, which is a key cytokine involved in initiating Th1-type CD4^+^ T-cell responses (111). These studies not only support the view that some β-glucans could modulate how host immune system recognizes and responds to antigens but also provide evidence that β-glucan-induced antigen recognition and immune responses could further enhance the adaptive immunity and immune memory.

### β-Glucan-Based Nanoparticle Systems

Cancer immunotherapy with nanoparticles is an emerging area with significant promise in oncology. Research into glucan molecules as specific immune modulator has inspired the development of glucan-based multifunctional nanoparticle systems for targeted delivery of glucan combined with therapeutic agents to immune cells, with the anticipation that such approach could amplify glucan-mediated immune activation for more effective antitumor immunotherapy.

The particulate yeast-derived BG36 glucans are hollow, porous 2–4 µm spheres with an outer shell capable of mediating uptake by phagocytic cells *via* dectin-1 activated pathway. Therefore, high payload of therapeutic agents such as DNA, siRNA, protein/peptide, and small molecules could be encapsulated into the particles using a core-polyplex and layer-by-layer synthetic strategies. Such synthetic particulate yeast BG36-based particle system benefits from a high payload of therapeutics encapsulation and targeted interaction with macrophages. An *in situ* layer-by-layer synthesis of DNA-caged BG36 particles was shown to not only effectively protect the caged DNA from degradation but also facilitate the systemic delivery of DNA content to macrophages *in vivo* (112–114).

BG34 glucans are soluble molecules with multiple aldehyde and hydroxyl groups, which provide tremendous opportunity for constructing multifunctional nanoparticle systems. In our studies, we developed technologies to enable BG34 conjugation to the outer shell of carbon nanotubes of 80–100 nm in length and 10–20 nm in diameter. Carbon nanotubes can mediate near infrared trigger imaging and thermal ablation of target cells or tissues; for these reasons, they have been used for image-guided therapy. The elongated shape of nanotube can adapt an ordered alignment within vasculature, leading to fold-increases of blood circulation half-life as compared to spherical nanoparticles of similar sizes. Our BG34-conjugated carbon nanotubes were also conjugated with Fe_3_O_4_ nanoparticles to enable the magnetic resonance contrast effect (104). *In vitro* studies have demonstrated that BG34- and Fe_3_O_4_-conjugated carbon nanotubes can mediate excellent MRI-guided imaging of target cells and dose-dependent thermal ablation of PMJ2R macrophages upon near infrared laser irradiation, suggesting the potential of this nanotube system for MRI-guided therapeutic modulation in targeted cells/tissues. The i.v. administration of BG34- and Fe_3_O_4_-conjugated carbon nanotubes in mice bearing 4T1 mammary tumors resulted in nanotube accumulation in 4T1 tumors. Pathological analysis of 4T1 tumors showed that tumor-infiltration CD206^+^F4/80^+^ TAMs stained positive for iron, suggesting that BG34- and Fe_3_O_4_-conjugated carbon nanotubes can efficiently deliver imaging and therapeutic components into macrophage accumulated within TME. Upon accumulation of BG34- and Fe_3_O_4_-conjugated carbon nanotubes in TME as observed by MR imaging, three cycles of near infrared laser irradiation were applied directly to tumors resulting in a 24.5% reduction of macrophages in TME and 15% reduction of MDSCs in circulation as compared to untreated mice or mice treated with non-targeted carbon nanotubes. More importantly, the nanotube-mediated reduction in macrophages and MDSCs was associated with a significant reduction of lung metastasis (104).

## Clinical Trials of β-Glucan for Cancer Immunotherapy

Since β-glucan therapy has achieved great success in preclinical animal models, many efforts are now underway to determine its clinical therapeutic efficacy. Currently, there are multiple β-glucan-based clinical trials in cancer immunotherapy (summarized in Table 2), many of which utilize β-glucan in conjunction with Abs or tumor vaccines.

**Table 2:** Clinical trials of β-glucans.

Strategy	Proposed mechanism of action	Intervention		Conditions	Status	Clinical trial identifier
		Beta-glucan	Immunotherapy			
Cancer vaccine plus oral beta-glucan	Iincrease number of immune cells to boost efficacy of cancer vaccine	Yeast-derived particulate beta-glucan	1650-G cancer vaccine 1650-G	Lung cancer	Phase I	NCT018293 73
Orally administered beta-glucan as single agent	Prime neutrophil complement receptor 3 (CR3) mediate CR3-DCC, change myeloid-derived suppressor cell, T cell functions, alter macrophage phenotype (M1 vs. M2)	Yeast-derived particulate beta-glucan		Non small cell lung cancer	Phase I	NCT006820 32
Orally administerted beta-glucan as single agent	Affect leukocyte number and function	Yeast-derived beta-glucan		Immunologic deficiency syndrome	Phase I	NCT017278 95
Soluble beta-1,3/1,6-glucan as single agent	Boost immune system to reduce mucositis upon completion of chemotherapy/radiotherapy and after oral treatment with beta-1,3-1,6-glucan or placebo	Soluble beta-1,3/1,6-glucan	Radiotherapy and chemotherapy	Oral mucositis in head and neck cancer patients	Phase II	NCT002890 03
Orally administatered yeast β-glucan plus tumor-specific antibodies	Specific antibody mediate complement activation of tumor cells and beta-glucan induces leukocyte killing of the complement-activated tumor cells	Yeast-derived beta-glucan	Anti-GD2 monoclonal antibody (mAb) 3F8	Metastatic neuroblastoma	Phase I	NCT004921 67
Orally administatered yeast β-glucan plus tumor-specific mAb	Beta-glucan may increase the effectiveness of rituximab by making cancer cells more sensitive to the mAb	Yeast-derived beta-glucan	Rituximab	CD20^+^ lymphoma or leukemia or posttransplant lymphoproliferati ve disease	Phase I	NCT000870 09
Orally administatered yeast β-glucan plus immunological	Boost overall immune system and leukocyte function	Yeast-derived beta-glucan	OPT-821	High-risk neuroblastoma	Phase I/II	NCT009115 60
Orally administered beta-glucan plus mAb rituximab	Increase efficacy of mAb	Soluble yeast-derived beta-glucan	Rituximab, COP/CHOP	Non-hodgkin’s lymphoma		NCT005337 28
Beta-glucan MM-10-001 as single agent	Changes in natural killer cell activation and functional activity, cytokine profiling, and clinical benefit	Beta-glucan MM-10-001		Locally advanced or metastatic non-small cell lung cancer	Phase I	NCT008570 25
Beta glucan PGG plus alemtuzumab and rituximab	PGG stimulate the immune system in different ways and help monoclonal antibodies kill CLL cells	Beta-glucan PGG	alemtuzumab and rituximab	CLL	Phase I/II	NCT012693 85
Beta-glucan in combination with standard antibody treatment and chemotherapy for breast cancer	SBG can enhance standard immunotherapy and chemotherapy for breast cancer	Beta-glucan SBG	Standard antibodies and chemotherapy	Breast cancer	Phase I/II	NCT005333 64
Beta-glucan PGG plus rituximab	PGG enhance immunotherapy using mAb	Beta-glucan PGG	Rituximab	NHL	Phase II	NCT020861 75
Beta-glucan PGG plus mAb BTH1704 and Chemotherapy gemcitabine	PGG triggers leukocyte-mediated cytotoxic response toward tumor cells, is anticipated to enhance immunotherapy using mAb targeting mucin 1 and gemcitabine	Beta-glucan PGG	BTH mAb and Gemcitabine	Advanced Pancreatic Cancer	Phase I b	NCT021324 03
Beta-glucan SBG as single agent	Immune potentiating and antitumor activity	Beta-glucan SBG		Advanced solid tumor	Phase I	NCT019105 97
Beta-glucan and other biological therapy plus mAb 3F8	3F8 can locate tumor cells and either kill them or deliver tumor-killing substances to them without harming normal cells. Biologicals increase the effectiveness of 3F8 by making tumor cells more sensitive to the antibody	Beta-glucan, isotretinoin, and sargramotism	mAb 3F8	Neutoblastoma	Phase II	NCT000892 58

Clinical experience to-date suggests that combination cancer immunotherapy is the future of cancer therapies for many cancers. Combined immunotherapy utilizing β-glucan and antitumor Abs represents one approach of breaking tumor-induced tolerance to enhance cancer immunotherapy. Combination β-glucan plus Abs therapy offers several unique advantages over other immunotherapeutic approaches. First, this approach uses humanized tumor-specific mAbs to target tumors and sensitize tumor cells for β-glucan treatment and, therefore, does not rely on the patients’ own immune responses. Second, in addition to mAbs listed in Table 2, any antitumor mAbs capable of activating the complement system can be used in combination with β-glucan. Recently, a combination therapy using β-glucan and mAbs targeting immune checkpoint molecules such as PD-1 and PD-L1 has been investigated in preclinical models with promising antitumor efficacy, and is anticipated to be translated into a phase I clinical trial (115, 116). Third, the clinical usage of checkpoint blockade Abs continues to grow and will be incorporated into the standard of care for multiple cancer types. Increases in the number of immune-modulating Abs offer more opportunities to design versatile combinations with β-glucan therapy. Fourth, this approach can also be used in synergy with most tumor vaccines as long as the tumor vaccine can elicit antitumor humoral responses and the Abs can bind to tumor cells to activate the complement system. For the tumor vaccines that elicit potent cytotoxic T cell responses along with humoral responses, combining therapy with β-glucan can add additional efficacy of innate and adaptive immune responses due to a potential role of β-glucan as an immune adjuvant.

## Future Perspectives

Although β-glucan appears to stimulate antitumor immune responses *via* direct interaction with macrophages, it may induce interaction with other type of immune cells. For example, in addition to stimulating macrophages, particulate yeast-derived BG36 β-glucan could also stimulate DCs to secrete pro-inflammatory cytokines including IL-12, TNF-α, and IL-6. The glucan-induced production of IL-12 and TNF-α was not altered in dectin-1 KO DCs, suggesting that glucan-mediated activation of DCs were not dectin-1-dependent (117). Another example was bacteria-derived BG36 glucan, Curdlan, which could directly convert T_reg_ into Th17 effector cells *in vitro*. It remains to be seen whether and how β-glucan-type molecules could affect phenotype and function of a variety of immune cells, and whether and how β-glucan-mediated immune stimulation involves other cellular receptors and pathways.

β-glucans with different structures appear to stimulate antitumor responses in completely different manners, and these detailed molecular mechanisms need to be further investigated. *In vivo* administration of oat-derived BG34 glucan could stimulate production of pro-inflammatory cytokines in TME, and the protective immunity was associated with a significant reduction in tumor-infiltrated granulocytes (105). In contrast, *in vivo* administration of soluble yeast-derived BG36 glucan did not induce any pro-inflammatory cytokines in TME, and the protective immunity was associated with a significant increase of tumor-infiltrating neutrophils that are primed for CR3-DCC (98). These results suggest that β-glucans from different sources with different glycosidic linkages, Mws, solubility, and administrative routes exhibit different mechanisms of actions. Identifying the structural-function relationship of β-glucan will not only enhance our understanding of β-glucans as immune modulator to stimulate innate and adaptive antitumor immune responses but also help to perform well-designed clinical trials to assess antitumor efficacy of β-glucans and related biomolecules.

A recent study showed that CD11b could differentially regulate TLR4-induced signaling pathways in DCs but not in macrophages (118). This study demonstrated CD11b as a key mediator of the adjuvant effect of LPS in DCs, but not in macrophages. Furthermore, the CD11b dependency is not the result of a GM-CSF-mediated cell-programming effects but involves endosomal TRIP-dependent pathways in DCs. These observations suggested that CD11b-mediated effects are likely to be restricted to a specific stage in the macrophage vs. DC lineage differentiation process. Since multiple studies have demonstrated specific binding of BG34 (105) and BG36 (91) with CD11b, these observations provided strong rationale for investigating whether these glucans can specifically affect subtypes of CD11b^+^ myeloid cells or a particular stage of the CD11b^+^ macrophage vs. DC lineage differentiation process. Identification of β-glucans capable of promoting this specific differentiation pathway may lead to novel clinical applications in cancer and other immune-mediated diseases.

New emerging data regarding the modulatory effect of β-glucan on regulatory cells such as T_reg_, TAMs, and MDSCs are of great importance (92, 98, 119). Currently, the main challenge of cancer immunotherapy is to inhibit the tumor-induced suppressive mechanisms and establish a TME, which is favorable for developing therapeutically efficacious antitumor immune responses. It is becoming clear that T_reg_, TAMs, and MDSCs are major contributors to the immune inhibitory signals limiting the adaptive antitumor immunity. Identification of the molecular mechanisms and inhibitory effects of β-glucan on these regulatory cell subsets is a critical step to develop specific immune modulator for targeted TME modulation to enhance cancer immunotherapy.

Careful selection of β-glucans is essential. In this regard, oat-derived BG34 β-glucan offers several advantages for developing high-quality clinical investigations to enhance cancer immunotherapy. First, BG34 can be highly purified to remove all possible peptide and protein complex contaminants, while BG36 glucans isolated and purified from yeast or mushroom always have undesirable peptide or protein conjugates (~5–30% protein) (120). Second, oat BG34 glucans are derived from cell-wall of plant instead of microorganism (fungi or bacteria). This allows the preparation of BG34 glucans completely free of endotoxins. Third, BG34 glucans exhibit linear and flexible chain structure in solution, allowing for isolating BG34 glucans into fractions with same chemical structures with specific Mws (106). Since the interaction of β-glucan-type molecule with murine and human leukocytes is greatly affected by glucan-associated impurities, endotoxin contaminants, and broad Mw distribution, the abovementioned features of BG34 glucans make them excellent candidates for therapeutic development.

Finally, despite potential therapeutic efficacies of combining β-glucan and various immune-modulating Abs, challenges remain ahead of achieving success with this approach. The anti-inflammatory TME milieu and the overexpression of membrane complement regulatory proteins can limit tumor-infiltrating immune cell subsets as a result of β-glucan priming. Over-expression of CD55 on SKOV-3 tumors can significantly impair complement activation and C5a release within the tumors (121), which also limits the β-glucan-primed immune cells infiltration into tumors. Inefficient iC3b deposition on tumor cells and C5a release within tumor tissue may make combination approach with β-glucan and Abs less efficacious. Therefore, additional strategies such as the use of exogenously administered pro-inflammatory cytokines or agents to induce inflammatory cytokine productions in TME may be added to overcome these obstacles. A cocktail of tumor-directed Abs targeting multiple tumor-associated antigens can also be used as a potential means to amplify complement activation and deposition of iC3b onto tumor cells to improve β-glucan-mediated therapeutic efficacy.

## Author Contributions

MZ is first and corresponding author. JK and AH are co-corresponding authors.

## Conflict of Interest Statement

The authors declare that the research was conducted in the absence of any commercial or financial relationships that could be construed as a potential conflict of interest.
